# Effect of Sodium *C*-Tetra(propyl)resorcin[4]tetrasulfonate (Na_4_PRA) on Antituberculosis Drugs as Seen by Diffusometry and NMR Spectroscopy

**DOI:** 10.3390/ijms27177657

**Published:** 2026-08-26

**Authors:** Edilma Sanabria, Ana C. F. Ribeiro, Ana M. T. D. P. V. Cabral, Mauricio Maldonado

**Affiliations:** 1 Grupo GICRIM, Programa de Investigación Criminal, Universidad Manuela Beltrán, Avenida Circunvalar No. 60–00, Bogotá 111321, Colombia; edilma.sanabria@docentes.umb.edu.co; 2 CQC-IMS, Department of Chemistry, University of Coimbra, 3004-535 Coimbra, Portugal; 3 Faculty of Pharmacy, University of Coimbra, 3000-548 Coimbra, Portugal; acabral@ff.uc.pt; 4 Departamento de Química, Facultad de Ciencias, Universidad Nacional de Colombia, Sede Bogota, Carrera 30 No. 45-03, Bogotá 111311, Colombia; mmaldonadov@unal.edu.co

**Keywords:** *C*-tetra(propyl)resorcin[4]tetrasulfonate, ethambutol dihydrochloride, isoniazid, NMR, supramolecular complexes, transport properties

## Abstract

The present study investigates the physicochemical behavior of the first-line anti-tuberculosis drugs isoniazid (INH) and ethambutol, in the form of dihydrochloride (E·(HCl)_2_), in aqueous solutions containing the synthetic macrocyclic resorcinarene, *C*-tetra(propyl)resorcin[4]tetrasulfonate (Na_4_PRA) at 298.15 K. Taylor dispersion experiments were conducted to determine the ternary diffusion coefficients of these systems, offering valuable insights into their transport properties. Non-zero cross-diffusion coefficients ($D_{12}$ and $D_{21}$) demonstrate significant coupled transport, collectively indicating interactions between these antibiotics and the resorcinarene host. This behavior is highly consistent with the formation of a host–guest complex. These diffusion measurements were complemented by NMR spectroscopy, which confirmed the formation of host–guest complexes between the respective drugs and this resorcinarene, Na_4_PRA.

## 1. Introduction

*Mycobacterium tuberculosis* is an aerobic, intracellular bacterium (also called Koch’s Bacillus after its discoverer, Robert Koch, in 1882) that causes tuberculosis. According to the World Health Organization, tuberculosis (TBS) was the second leading infectious cause of death worldwide in 2022, after COVID-19 [1]. However, it has quickly gained ground, now becoming the leading cause of death according to the World Health Organization [2]. This disease can affect various organs, primarily the lungs, brain, kidneys, spine, lymph nodes, heart, and joints. These organs can be affected simultaneously and even in conjunction with other diseases [3]. It also occurs in animals, both domestic and wild, in captivity. In Nigeria, for example, it has been found in pigs, goats, camels, and cattle. The bacteria are not the same, but they can be transmitted to humans through contact or, for example, by drinking raw milk, causing a disease very similar to human tuberculosis [4]. Tuberculosis can be classified as pulmonary (PTB) or extrapulmonary (EPTB) [5]. The primary form is the most prevalent and highly transmissible. Transmission occurs via respiratory droplets expelled during the persistent cough characteristic of the disease, as well as through vocalization, singing, or sneezing by an infected individual. Extrapulmonary tuberculosis, in contrast, can affect multiple organs and systems, including the central nervous system, skin, bones, joints, the genitourinary tract, abdomen, lymph nodes, and the pleura [6]. The clinical presentation of tuberculosis varies according to the organ affected; however, common symptoms include fever, general malaise, and weight loss. Because these symptoms are too general, they may hinder clinical suspicion and contribute to delayed diagnosis [7]. Several diagnostic methods are available for tuberculosis, including the C-reactive protein test, the molecular test of the tongue swab, the digital chest X-ray, the cough sound signatures [8], sputum culture, and blood test [9]. The treatment of tuberculosis primarily involves two types of drugs: bactericidal agents, which kill the bacterium, and bacteriostatic agents, which inhibit bacterial growth by preventing cell wall formation [10]. The medications commonly used to treat tuberculosis are generally classified as first-line or second-line [11]. First-line drugs are more standardized, provide better disease control, are less harmful to people, and are more accessible due to their low cost. This group includes isoniazid, rifampicin, rifapentine, pyrazinamide, and ethambutol. Second-line drugs are used when first-line drugs are ineffective or cannot be administered. They tend to be less effective, more expensive, and their treatment is longer and more complex, often with higher toxicity. Examples include levofloxacin, moxifloxacin, p-aminosalicylic acid, cycloserine, ethionamide, protionamide, streptomycin, amikacin, and Kanamycin [11]. These drugs have developed resistance over time [12], which is why, in recent times, there has been a need to implement multi-drug treatment in phases (never monotherapy). For example, the first phase typically uses four medications to eliminate resistant bacterial mutants and reduce the overall bacterial load, while the second phase uses two drugs to consolidate treatment [13]. This approach works because each drug targets a different part of the bacterium, allowing it to be attacked from multiple angles to eradicate it or at least control its growth. Isoniazid, for example, has a bactericidal effect because it inhibits the synthesis of mycolic acids, which are essential for the formation of the bacterial cell wall. Rifampicin blocks bacterial RNA polymerase, preventing bacteria from producing proteins. Pyrazinamide, a prodrug of pyrazinoic acid currently used as a first-line therapy for tuberculosis, possesses unique properties, such as excellent sterilizing activity and multiple mechanisms of action. This drug is active against intracellular microorganisms, particularly within the acidic pH environment of macrophages. Ethambutol is bacteriostatic because it acts by inhibiting the synthesis of arabinogalactan, an essential component of the cell wall [14]. In addition, the use of these drugs faces several challenges. Conventional treatment is often prolonged and expensive. Furthermore, many therapeutic agents suffer from poor bioavailability, driven by unfavorable physicochemical properties such as extremely high or low water solubility, high permeability, and rapid metabolism that prevent maintaining adequate cellular concentrations. Additionally, some drugs exhibit a low affinity for their intended targets [15].

Considering these challenges, the present study establishes its foundation by investigating the physicochemical behavior of the first-line antitubercular drugs isoniazid (INH) and ethambutol (E·(HCl)_2_). Indeed, the literature shows that isoniazid poses significant hurdles for conventional controlled-release systems; its high water solubility accelerates release rates in aqueous media, leading to rapid concentration peaks [16,17] that can cause hepatotoxicity and consequently reduce patient compliance. While ethambutol has been less explored in this context, a similar or perhaps more pronounced issue is anticipated. Ethambutol is administered as a hydrochloride salt (E·(HCl)_2_), a form that drastically increases its aqueous solubility. Furthermore, although these drugs are well-established in clinical practice, a fundamental evaluation of their transport behavior in aqueous solutions containing different carriers remains lacking—an area where, to the best of our knowledge, a critical gap in the literature exists.

The development of host molecules, such as cyclodextrins, crown ethers, calixarenes, and resorcinarenes, has become a focal point in this field owing to their capacity to selectively recognize and interact with target molecules [18,19]. In the present work, resorcinarenes stand out as the compounds of choice because of their unique properties. These bowl-shaped macrocyclic compounds, formed by the condensation of resorcinol and aldehydes [20], are firmly positioned in the preclinical [21,22] and translational research phases.

Structurally, they feature a defined cavity, an upper rim, and a lower rim, all of which can be selectively functionalized. Specifically, the lower rim can be tailored with hydrocarbon chains to modulate the macrocycle’s hydrophobicity, while the upper rim can be modified with various functional groups—such as sulfonate [20], amino, or alkynyl moieties [23] —to impart water solubility [24,25] a critical requirement for effective drug delivery systems (DDS) [18] While not currently used in active clinical care, resorcinarenes are being heavily engineered for key biomedical applications, including advanced DDS [26], experimental antimicrobial and antitumor therapies [26], bioimaging [27] and separation science [28].

While cyclodextrins have traditionally dominated the field of macrocyclic drug carriers, resorcinarenes offer distinct advantages in terms of chemical versatility and structural adaptability. Unlike the relatively rigid and neutral cavities of cyclodextrins, resorcinarenes can be tailored with specific functional groups—such as the negatively charged sulfonate moieties on the upper rim of sulfonated resorcinarenes. This functionalization creates a highly electron-rich, biomimetic cavity capable of driving strong electrostatic, host-guest, and cation-π interactions. Consequently, resorcinarenes represent a highly promising and tunable platform for the transport of polar and ionized drugs, unlocking encapsulation mechanisms that are unavailable to conventional cyclodextrin matrices.

The aim of the present work is to evaluate the host-guest complexation of these first-line drugs with a water-soluble resorcinarene, specifically sodium *C*-tetra(propyl)resorcin[4]tetrasulfonate (Na_4_PRA). This approach takes advantage of the macrocycle’s dual properties: a hydrophobic interior (inside the cone) and a hydrophilic exterior, which allows it to access cellular regions that the drugs alone could not reach, thereby enhancing their effectiveness. To the best of our knowledge, no data have yet been reported on the ternary mutual diffusion coefficients of INH and E·(HCl)_2_ in aqueous solutions containing Na_4_PRA at 298.15 K. To fill this literature gap, the present work provides comprehensive diffusion coefficient data for aqueous E·(HCl)_2_ (or INH) + Na_4_PRA ternary systems. These measurements, conducted at 298.15 K via the Taylor dispersion technique, are complemented by NMR analysis to provide a deeper insight into the systems’ behavior. The investigated concentration range (0.0 to 10.0 mmol dm^−3^) for both INH and (E·(HCl)_2_) was selected based on a carefully optimized compromise between instrumental requirements and pharmaceutical relevance, justified by two main reasons. First, high-precision transport techniques, such as the Taylor dispersion method, strictly require solute concentration gradients in the millimolar range or higher to achieve a satisfactory signal-to-noise ratio and ensure accurate, reproducible diffusion data. Second, from a biopharmaceutical perspective, while a concentration of 10 mmol dm^−3^ does not reflect the highly diluted conditions of systemic blood circulation, it accurately mimics the local, concentrated microenvironment established at the precise moment of drug dissolution. However, by extrapolating to infinite dilution ($X_{INH(or\;E\cdot (HCl)2)}\to 0$), it remains possible to estimate transport behavior under the highly diluted conditions typical of systemic circulation.

## 2. Results

### 2.1. Synthesis and NMR Characterization of Na_4_PRA

**Scheme 1:**
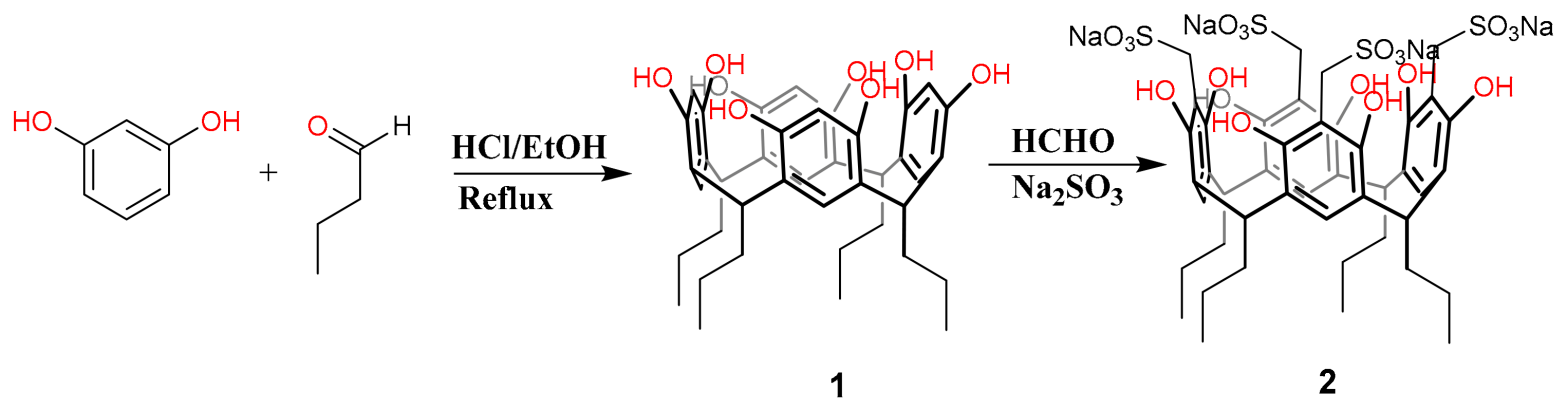
Stages of the synthesis of calix[4]resorcinarene, Na_4_PRA (**2**).

For the development of this study, the first stage consisted of the synthesis of *C*-tetra(propyl)calix[4]resorcinarene (**1**), which was done by direct reaction of butyraldehyde with resorcinol using a mixture of ethyl alcohol-H_2_O as the solvent. The reaction was done stirred and heating to 80 °C for 12 h, as described previously [20] (Scheme 1). Once this reaction time had elapsed, a solid precipitate was formed. The derivative was characterized using spectral techniques, including IR, ^1^H-NMR, and ^13^C-NMR. The resorcinarene (**1**) had been previously synthesized, and our spectroscopic data agreed with those reported [29]. The *crown* conformation of the product was verified by the ^1^H-NMR spectra: Only one resonance signal was observed for the hydroxyl protons of 1 at 8.90 ppm, as well as other signals in the spectrum; therefore, the ^1^H-NMR spectra of compound **1** were consistent with the presence of the *crown* conformer.

For the second stage of the synthesis, the sulfonation of tetra(propyl)calix[4]resorcinaren was carried out by direct reaction with formaldehyde and sodium sulfite solution, as shown in Scheme 1. The obtained product **2** was characterized using spectroscopic techniques, including IR, ^1^H-NMR, and ^13^C-NMR. Initially, the formation of sulfonated tetra(propyl)calix[4]resorcinarene (**2**) was confirmed with the appearance of the characteristic S=O band at 1051 cm^−1^ and C-S band at 606 cm^−1^. In the ^1^H-NMR spectra, the appearance of new aliphatic protons at 3.87 ppm, assigned to the methylene bridge between the sulfonate group and the resorcinol ring (Ar-CH_2_-SO_3_Na), and the disappearance of the signal at 6.17 ppm confirm the sulfomethylation reaction. In addition, the singlet signal observed at 7.23 ppm confirms that the macrocycle conformation is retained; that is, the conformation of macrocycle **2** is of the *crown*-type (Scheme 1).

Additionally, the ^1^H-NMR spectrum of the synthesized sulfonated calix[4]resorcinarene (Na_4_PRA, **2** in Scheme 1) in DMSO-d_6_ was consistent with the presence of a single conformer. In this way, in the ^1^H-NMR spectrum of compound **2**, two signals that confirmed the *crown* conformation could be seen: a singlet signal at 9.70 ppm for the hydroxyl groups and another singlet signal in the aromatic zone for the proton *meta* to OH at 7.34, these signals are consistent with other analog macrocycles in the *crown* conformation. Finally, the number of signals observed in the ^13^C-NMR spectrum confirmed the structure of compound **2**. These results indicate that during the sulfonation reaction, this conformation is retained in solution.

### 2.2. NMR Studies of Isoniazid (INH) and Ethambutol (E·(HCl)_2_ in Aqueous Solutions in the Absence and Presence of Na_4_PRA

**Figure 1:**
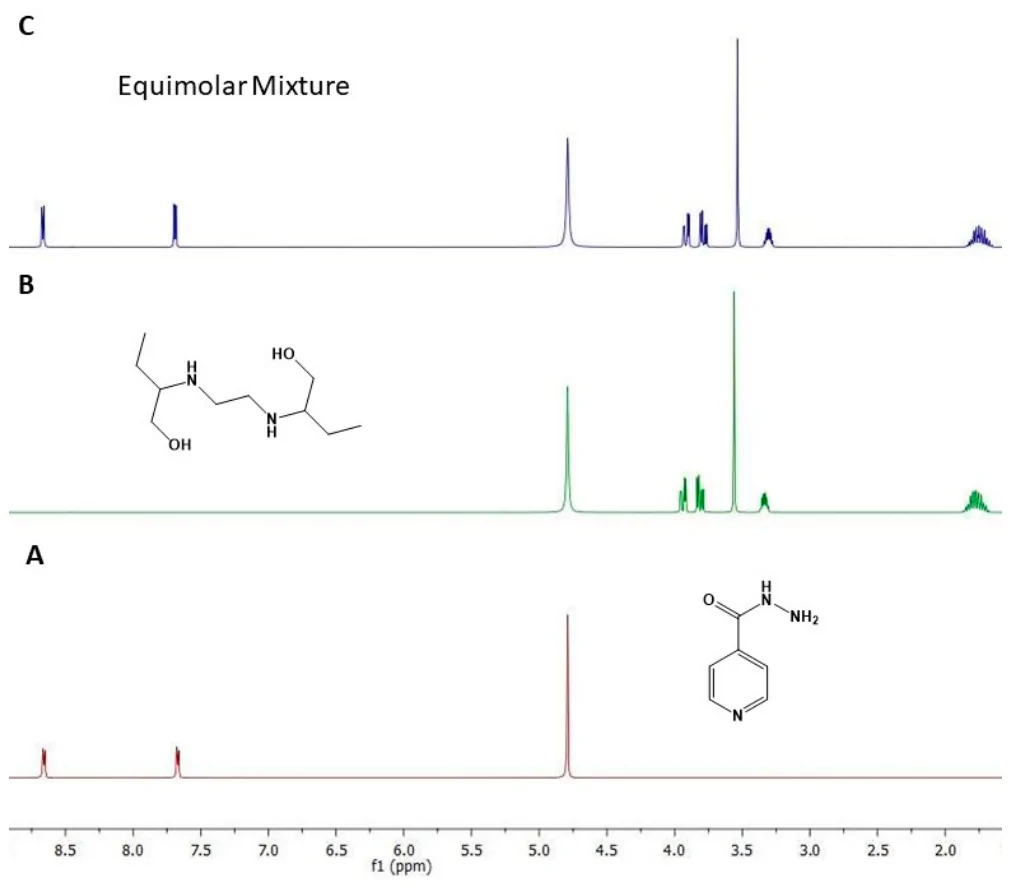
^1^H-NMR of INH in red (spectrum (**A**)), E·(HCl)_2_ in green (spectrum (**B**)), equimolar mixture in blue (spectrum (**C**)).

The next stage of this study involved evaluating the molecular interactions between the compounds INH and E·(HCl)_2_ in aqueous media using NMR spectroscopy. First, as described in the experimental section, the ^1^H-NMR spectra of INH and E·(HCl)_2_ were obtained independently to establish the signals that would allow for the evaluation of any changes that might occur. The ^1^H-NMR spectrum of E·(HCl)_2_ in deuterated water exhibited a total of six signals. Proton assignments were verified based on chemical shifts, integral values, and multiplicities, and were further confirmed by comparison with previously reported literature data. High-field signals were observed at 3.92, 3.82, 3.34, 3.56, 2.02, and 1.02 ppm finding that these patterns were consistent with the structure of the expected compound; the signal assignment is shown in Figure 1B [30]. On the other hand, the spectrum of INH in deuterated water showed a total of two signals at 8.66 ppm and another doublet signal at 7.67 ppm, which is consistent with what was previously reported [31] (Figure 1A). To evaluate the interaction between these two compounds, an equimolar mixture was prepared in deuterated water, observing that there is no noticeable interaction (Figure 1C).

**Figure 2:**
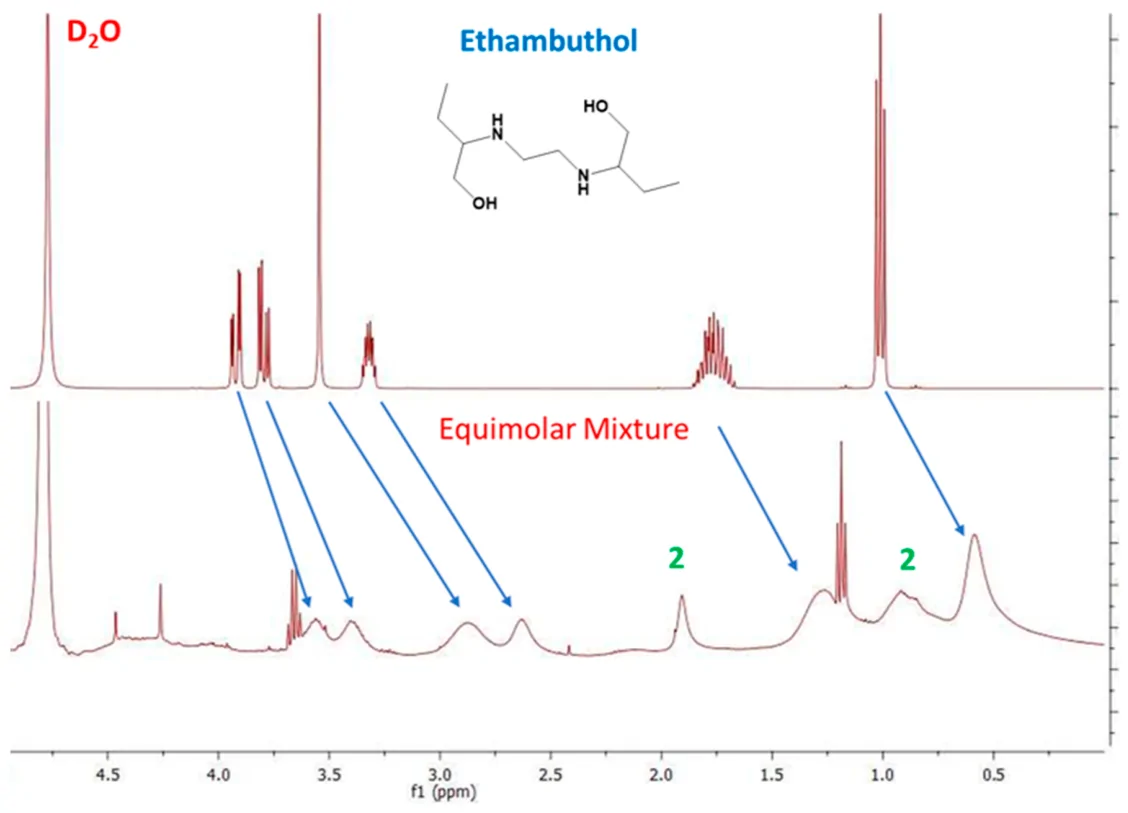
^1^H-NMR of E·(HCl)_2_ and equimolar mixture of resorcinarene (**2**) with E·(HCl)_2_.

In addition, the interaction between calix[4]resorcinarene (**2**, Scheme 1) and antituberculosis drugs INH and E·(HCl)_2_ was also evaluated via ^1^H-NMR in D_2_O. First, the interaction between compound **2** and E·(HCl)_2_ was evaluated by preparing an equimolar mixture of the two compounds in deuterated water. As can be seen in Figure 2, the signals in the spectrum of the mixture were compared with those of the spectrum of E·(HCl)_2_, and an important shift was observed in the corresponding signals in E·(HCl)_2_. In the equimolar mixture spectrum, the number **2** in green indicates the signals of sulfonated calix[4]resorcinarene (**2**), which are not greatly affected; however, the blue arrows highlight the changes observed for E·(HCl)_2_, which are significantly affected in the presence of the macrocycle. Considering this strong interaction observed between resorcinarene **2** and E·(HCl)_2_ the stability of the complex formed was evaluated through the variation in the ^1^H-NMR chemical shift in a single proton resonance in the E·(HCl)_2_ (singlet at 3.56 ppm), as a function of the molar ratio between **2** and E·(HCl)_2_, showing notable changes when varying the stoichiometric ratio (See experimental section). The protons most affected by the host-type interaction are the protons on the carbons attached to the amino group, observing changes with values in the chemical shift change (Δδ) of 0.68–0.91 ppm. Specifically, the signals at 3.56, 3.82, and 3.92 ppm, while the signals near the hydroxyl groups are less affected (signals at 1.02 and 1.76 ppm). These changes in the chemical shifts seem to be caused by a strong interaction between **2** and E·(HCl)_2_, mediated mainly by hydrogen bonds between the amino group (NH) and the oxygen atoms of the macrocycle, and by interactions with the π-electrons in the cavity of the macrocyclic system.

Similarly, the interaction between the resorcinarene **2** and INH was also evaluated by preparing an equimolar mixture under identical conditions. As mentioned previously, the INH heterocyclic system exhibits two signals for aromatic protons in deuterated water. Specifically, for protons at positions **2** and **6**, a signal is observed at 8.66 ppm, and for protons at positions **3** and **5** of the heterocyclic ring, a signal is observed at 7.67 ppm. In the equimolar mixture with resorcinarene **2**, these signals are affected, with shifts observed at 8.57 ppm (Δδ = 0.09 ppm) and 7.61 ppm (0.05 ppm), respectively. These results suggest two conclusions: First, the interaction of INH with resorcinarene **2** is less strong than the interaction of E (HCl)_2_ with **2**, and second, the interaction of the resorcinarene **2** cavity with INH occurs mainly with the nitrogens of the heterocyclic system.

The distinctly stronger interaction of E·(HCl)_2_ with ${\mathrm{Na}}_{4}\mathrm{PRA}$ compared to INH can be rationalized by analyzing their ionization behaviors and structural topologies. At the experimental pH (i.e., 4.20 ≤ pH ≤ 4.25), E·(HCl)_2_ exists predominantly as a dicationic species due to its protonated aliphatic amine groups ($pK_{a1}\approx 6.4$ and $pK_{a2}\approx 9.4$). This net positive charge promotes a highly favorable, strong electrostatic attraction with the four negatively charged sulfonate groups (${\mathrm{SO}}_{3}^{-}$) of the ${\mathrm{Na}}_{4}\mathrm{PRA}$ macrocycle. In contrast, INH remains in its neutral form under identical conditions ($pK_{a}\approx 1.8$ and 3.5), restricting its interaction with the carrier primarily to weaker hydrogen bonding and dipole–dipole forces.

Furthermore, conformational and steric factors play a decisive role. E·(HCl)_2_ is a highly flexible, open-chain aliphatic molecule. This structural adaptability allows it to undergo conformational adjustments, optimizing its spatial alignment to maximize electrostatic and van der Waals contacts with the macrocyclic host—including potential hydrophobic interactions between its butanol side chains and the hydrophobic core or propyl chains of ${\mathrm{Na}}_{4}\mathrm{PRA}$. Conversely, the rigid, planar aromatic ring of INH offers limited conformational freedom, preventing optimal structural nesting within the macrocyclic assembly. These combined electrostatic and steric contributions perfectly corroborate the more pronounced alterations observed in the diffusion coefficients and NMR chemical shifts for the E·(HCl)_2_-${\mathrm{Na}}_{4}\mathrm{PRA}$ system.

**Scheme 2:**
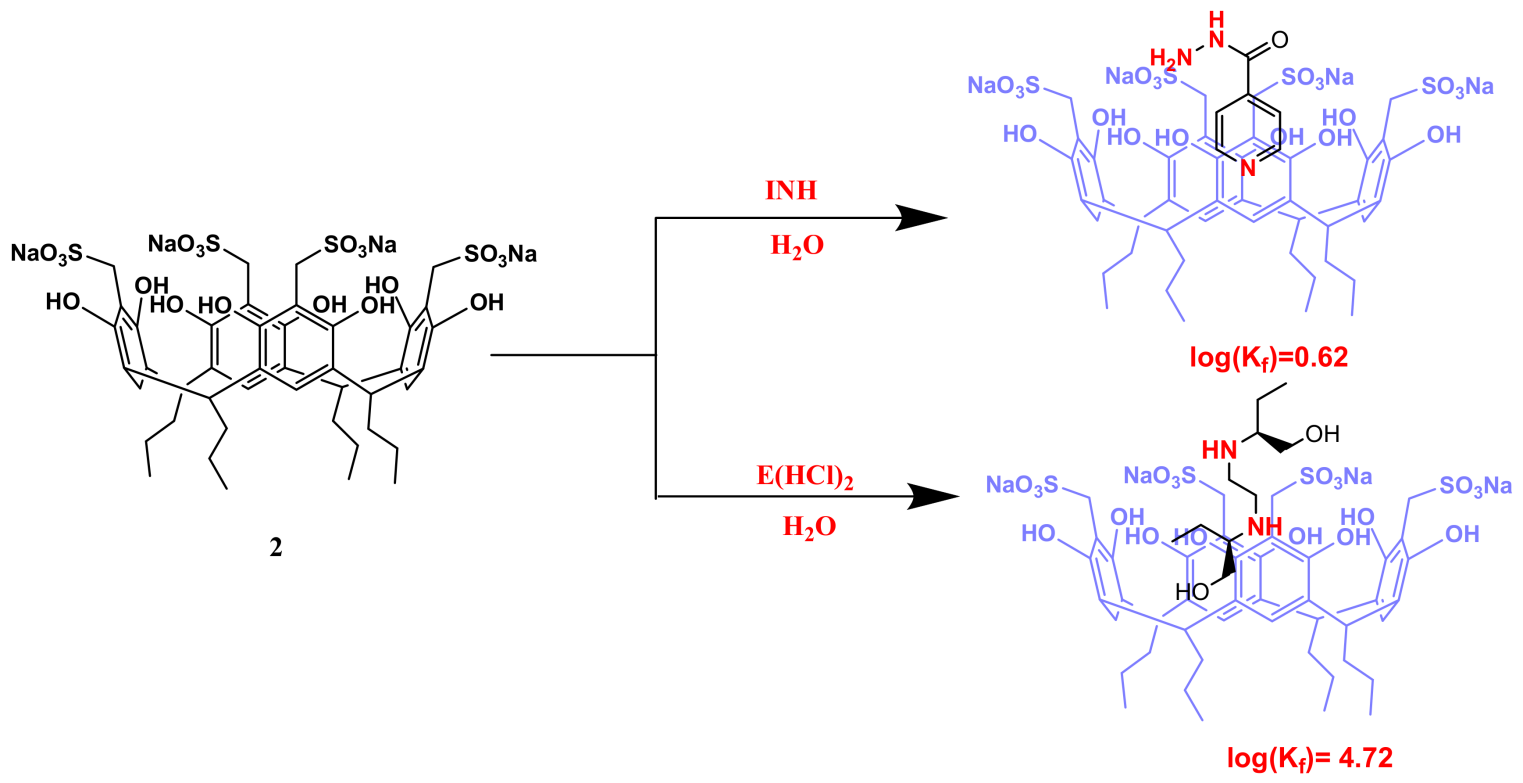
Binding constants (*K*_f_) for the complex of **2** with INH and E·(HCl)_2_.

Considering the strong interaction between resorcinarene **2** and E·(HCl)_2_, the stability of the complex formed was measured through the variation in the ^1^H-NMR chemical shift of a single proton resonance in the E·(HCl)_2_ (signal at 3.56 ppm), as a function of the molar ratio between E·(HCl)_2_ and resorcinarene **2**. The experimental data were fitted to the theoretical curves using the Hyp-NMR2008 program. In this way, a value for the formation constant (*K*_f_) was determined from the measured chemical shifts, with an error of 0.01 ppm, in the NMR spectra. Thus, for the complex between resorcinarene **2** and E·(HCl)_2_, a value of log (*K*_f_) = 4.72 was estimated. It is important to note that when the same experiments were performed on a mixture with a higher stoichiometric ratio between **2** and E·(HCl)_2_ (1:2 or 2:1), the same results were observed in the chemical shifts, which allows us to conclude that the host-guest complex is formed and, furthermore, that it has a 1:1 stoichiometry. In the same way, the interaction between **2** and INH was evaluated by estimating the affinity constant, which gave a value of log (*K*_f_) = 0.62. As can be seen in Scheme 2, the most probable interaction between **2** and INH occurs through the heterocyclic nitrogen of INH and the upper *rim* of **2**, since the most affected protons of INH are those at positions **2** and **6**. Considering the magnitudes of the constants obtained, it is found that their values are consistent with those obtained for similar systems [24].

### 2.3. Diffusion Coefficient Data

#### 2.3.1. Accuracy Validation of the Taylor Dispersion Technique

To assess the accuracy of the Taylor apparatus before proceeding to new systems, we measured the binary diffusion coefficients of aqueous KCl solutions at 298.15 K (Table 1). The resulting dispersion profiles (six replicates) were fitted using Equation (3) (Section 4.5.1). For these validation runs, the carrier and injection solution concentrations were $0.025\;{\mathrm{mol}\;\mathrm{dm}}^{-3}$ and $0.175\;{\mathrm{mol}\;\mathrm{dm}}^{-3}$, respectively, yielding a mean concentration of $0.100\;{\mathrm{mol}\;\mathrm{dm}}^{-3}$. This reference system serves as a reliable benchmark since its diffusion coefficients are accurately established in the literature [32,33].

Based on the close agreement between our experimental data and literature values (showing a deviation of 0.5%), combined with an experimental reproducibility of ±1%, the estimated overall uncertainty of 2% is highly reasonable and within acceptable standard limits.

#### 2.3.2. Aqueous INH (Component 1) + Na_4_PRA (Component 2) Solutions

The ternary diffusion coefficients for the systems comprising INH (component 1) and Na_4_PRA (component 2) in aqueous solutions are shown in Table 2. The main diffusion coefficients are generally reproducible within ±(0.015 × 10^−9^ m^2^ s^−1^), while the cross-diffusion coefficients show a reproducibility within approximately ±(0.040 × 10^−9^ m^2^ s^−1^). The pH of the aqueous isoniazid solutions measured was approximately 6.40, very close to the pH value of the water (6.41) used in the preparation of these solutions.

By analysis of Table 2, *D*_11_ values are considerably larger than the *D*_22_ values and decrease with the solute 1 fraction, defined as *X*_1_ = C_1_/(C_1_ + C_2_). At the limiting situations of *X*_1_ = 0 and *X*_1_ = 1 (where *X*_1_ represents the solute fraction of INH), the value of *D*_11_ corresponds, respectively, to the tracer diffusion coefficient of INH in Na_4_PRA and the binary mutual diffusion coefficient of aqueous INH at 0.0100 mol dm^−3^. Good agreement (3.2%) is observed between this last value for *D*_11_ obtained for INH (*D*_11_ = 0.850 × 10^−9^ m^2^ s^−1^), and the respective binary diffusion coefficient values previously obtained in other studies [34] (i.e., *D*_(INH)_ = 0.823 × 10^−9^ m^2^ s^−1^). Deviations of less than 3.2% are considered acceptable, as they fall within the typical uncertainties of the experimental method (which are generally ≤3%).

In the limit as *X*_1_ approaches zero, cross-coefficient values *D*_12_ are zero within experimental error, due to the inability of Na_4_PRA concentration gradients to drive coupled flows of INH in -free solutions of INH. However, the cross-coefficient *D*_12_ becomes very large and positive with increasing solute 1 fraction.

Regarding the behavior of *D*_21,_ the values are observed to be negative, reaching their most negative magnitudes as *X*_1_ → 0. In the other limit, *X*_1_ → 1, these cross-coefficient values are also close to zero, since INH concentration gradients cannot drive coupled fluxes of Na_4_PRA in Na_4_PRA -free solutions.

At $X_{2}=0$, the value of *D*_22_ corresponds to the tracer diffusion coefficient of ${\mathrm{Na}}_{4}\mathrm{PRA}$ in INH, which equals 0.626 × 10^−9^ m^2^ s^−1^. At $X_{2}=1$, the value of $D_{22}$ (0.600 × 10^−9^ m^2^ s^−1^) is expected to be close to the binary mutual diffusion coefficient of ${\mathrm{Na}}_{4}\mathrm{PRA}$ in aqueous solution at 0.008 mol dm^−3^ (that is, *D*_Na4PRA_ = 0.636 × 10^−9^ m^2^ s^−1^) [35].

#### 2.3.3. Aqueous E·(HCl)_2_ (Component 1) + Na_4_PRA (Component 2) Solutions

Table 3 shows the average experimental diffusion coefficients for aqueous E·(HCl)_2_ (component 1) + Na_4_PRA (component 2). The pH measurements were made on some of the ethambutol dihydrochloride solutions to assist interpretation of these results. For 0.001 mol dm^−3^ ≤ c_1_ ≤ 0.010 mol dm^−3^ and at 298.15 K, the pH values were in the range 4.20 ≤ pH ≤ 4.25. Ethambutol dihydrochloride acts as a weak diprotic conjugate acid ($pK_{a1}=6.35$ and $pK_{a2}=9.35$ at 25 °C). Consequently, in aqueous solutions, this drug remains predominantly in its diprotonated form (${\mathrm{EH}}_{2}^{2+}$, >99%) [36].

Main coefficients *D*_11_ and *D*_22_ give the molar fluxes of the E·(HCl)_2_ (1) and Na_4_PRA (2) driven by their own concentration gradient, respectively. Generally, these values are lower than the binary diffusion coefficients of aqueous E·(HCl)_2_ and Na_4_PRA, with deviations ranging from 7% to 36% [35,36].

Within the error limits of this method, *D*_12_ is approximately zero over this concentration interval. Conversely, *D*_21_ becomes increasingly negative as the E·(HCl)_2_ solute fraction decreases. However, in the limit *X*_1_ → 1, *D*_21_ is zero because E·(HCl)_2_ concentration gradients cannot drive coupled flows of Na_4_PRA in solutions that do not contain Na_4_PRA. Under those circumstances, *D*_22_ represents the tracer diffusion coefficient of Na_4_PRA in E·(HCl)_2_ solutions, and *D*_11_ must be an approximation of the binary mutual diffusion coefficient of E·(HCl)_2_ (4%) [36]. Similarly, *D*_12_ is zero in the limit *X*_1_ → 0. In this case, *D*_11_ is the tracer diffusion coefficient of E·(HCl)_2_ in Na_4_PRA solutions, and *D*_22_ also approaches the binary mutual diffusion coefficient of Na_4_PRA (4%) [35].

## 3. Discussion

The observed coupled diffusion behavior can therefore be rationalized based on electrostatic mechanisms. In binary Na_4_PRA solutions (Na_4_PRA + H_2_O), it is known that the diffusion of Na^+^ ions are higher than that of the PRA^4−^ anions macrocycle (*D*^0^_Na+_ = 1.334 × 10^−9^ m^2^·s^−1^ and *D*^0^_RA4−_ = 0.346 × 10^−9^ m^2^·s^−1^) [35]. As a result, a Na_4_PRA concentration gradient generates an electric field. This electric field slows down Na^+^ ions and accelerates PRA^4−^ ions so that both species migrate at the same velocity, ensuring zero electric current under electroneutrality conditions.

However, in ternary solutions of INH (1) + Na_4_PRA (2) + H_2_O, the electric field generated along the Na_4_PRA concentration gradient not only accelerates the PRA^4−^ ions but also induces the co-migration of isoniazid species (*D*_12_ > 0, Table 2), which interact with PRA^4−^, aiming to maintain the electroneutrality of the system. Considering that isoniazid is a weak base, and at neutral pH most of its molecules remain in their neutral form, this behavior can be explained based on the formation of supramolecular complexes. These structures arise from predominant non-covalent interactions between the drug and the hydrophobic cavity of the macrocycle—specifically, van der Waals forces and π-π stacking between the aromatic rings of both species. Secondary stabilization is provided by hydrogen bonding between the hydrazide group of INH (-CONHNH_2_-) and either the hydroxyl groups on the upper rim or the sulfonate groups on the lower rim of the resorcinarene, depending on the orientation of the molecule within the cavity. These findings are supported by NMR studies, which indicate that isoniazid preferentially enters the cavity pyridine-ring-first. This orientation leaves the more polar hydrazide moiety positioned at the macrocycle’s opening, where it is available for interactions with the sulfonate groups or the surrounding solvent.

Because INH is encapsulated within the macrocycle’s cavity, the resulting complex retains a −4 charge. Consequently, the Na_4_PRA concentration gradient generates an electric field that accelerates the transport of these complex species. This occurs because the Na^+^ ion lacks a high-mobility anionic counterpart and must effectively ‘drag’ the slow macrocycle to maintain electroneutrality. This mechanism provides a physical basis for the high positive values observed for the cross-diffusion coefficient, *D*_12_.

In the E·(HCl)_2/_Na_4_PRA system, the cross-diffusion coefficients, *D*_12_, are nearly zero within experimental uncertainty. This suggests that the electric field generated by the Na_4_PRA concentration gradient does not significantly influence E·(HCl)_2_ diffusion. These findings arise from the distinct chemical nature of this drug, which exists predominantly in its diprotonated form (${\mathrm{EH}}_{2}^{2+}$) in solution. Consequently, the formation of ion pairs—characterized by strong electrostatic interactions—occurs between the drug and the four negative sulfonate groups of Na_4_PRA. This leads to the formation of complexes that significantly reduce the effective net charge of the macrocycle. In this scenario, to maintain electroneutrality at the diffusion front under a Na_4_PRA concentration gradient, Cl^−^ ions originating from E (HCl)_2_ and Na^+^ ions diffuse nearly in tandem. This leaves the slower, more voluminous [E·(HCl)_2_ -PRA]^2-^ complex behind, as its reduced effective charge diminishes its response to the induced electric field, which leads to *D*_12_ = 0.

However, in both aqueous systems (INH/Na_4_PRA or E·(HCl)_2_)/Na_4_PRA), *D*_21_ < 0; that is, the concentration gradient of INH (or E (HCl)_2_) induces counter-current coupled flows of Na_4_PRA. This effect is most significant in highly concentrated solutions of Na_4_PRA. These observations can also be attributed to the association between INH (or E·(HCl)_2_) and these macromolecules, resulting in the formation of complexes in solutions. This complexation leads to a decrease in free Na_4_PRA concentration; consequently, to compensate for that local depletion, a counterflow of the macromolecule occurs. This effect is even more pronounced in the case of E·(HCl)_2_, indicating stronger intermolecular interactions. Support for these findings comes from NMR studies (Section 2.2).

**Figure 3:**
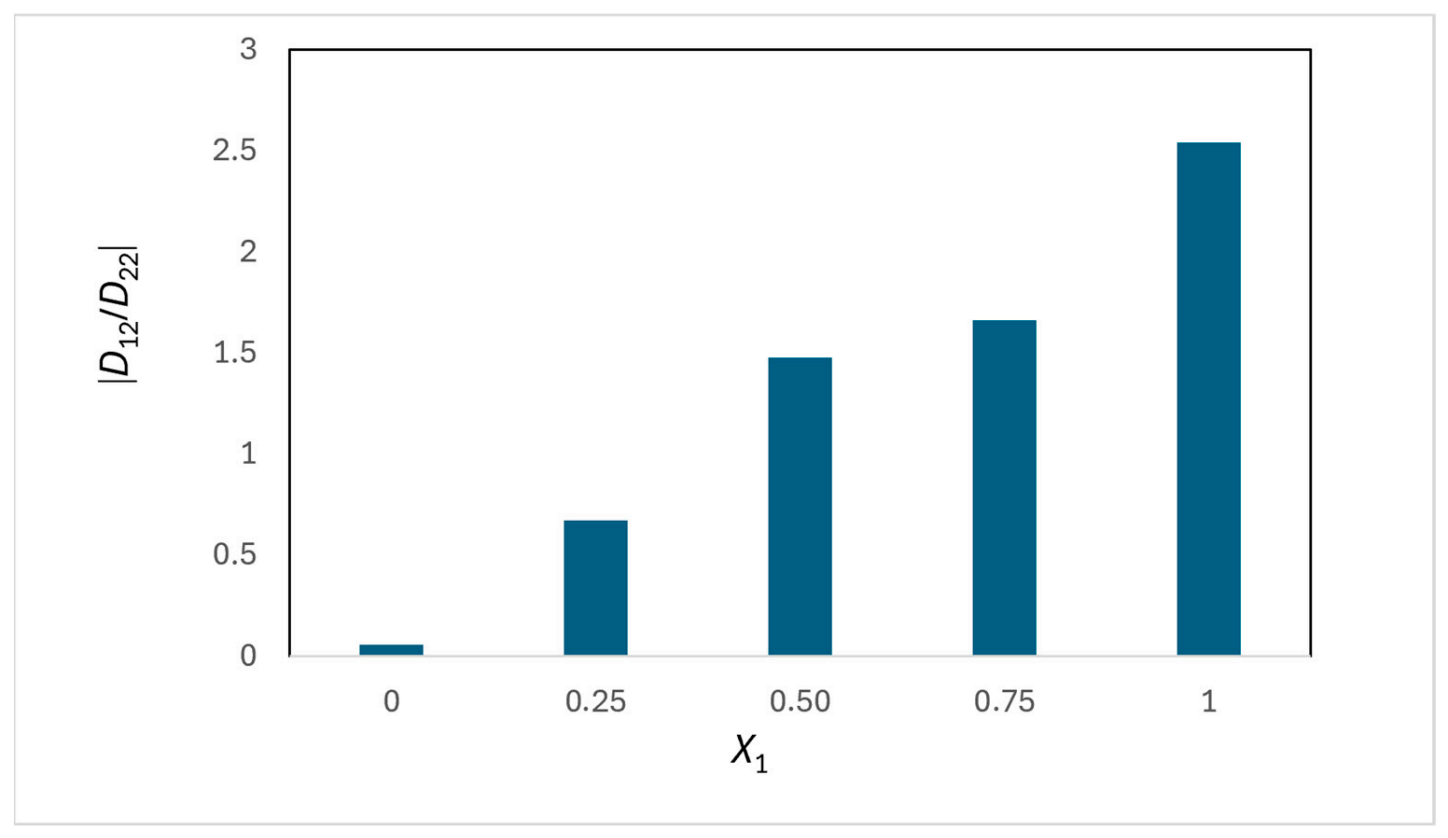
Estimation of moles of INH (component 1) transported for each mol of Na_4_PRA (component 2) for different values of molar fraction, *X*_1_:|*D*_12_/*D*_22_| represents the number of moles of INH co-transported for each mol of Na_4_PRA.

**Figure 4:**
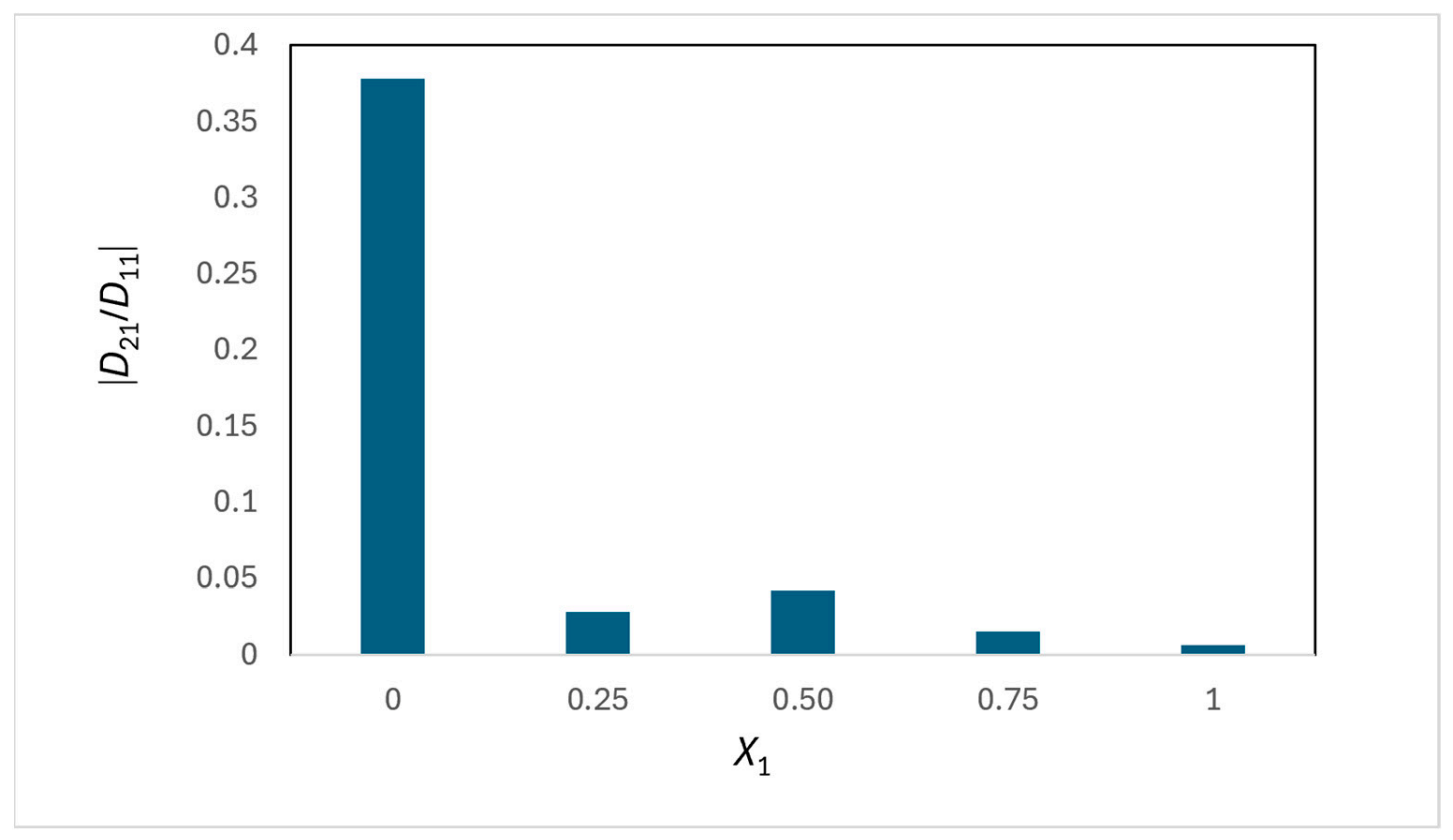
Estimation of moles of Na_4_PRA (component 2) transported for each mol of INH (component 1) for different values of molar fraction, *X*_1_:|*D*_21_/*D*_11_| represents the number of moles of Na_4_PRA counter-transported for each mol of INH.

**Figure 5:**
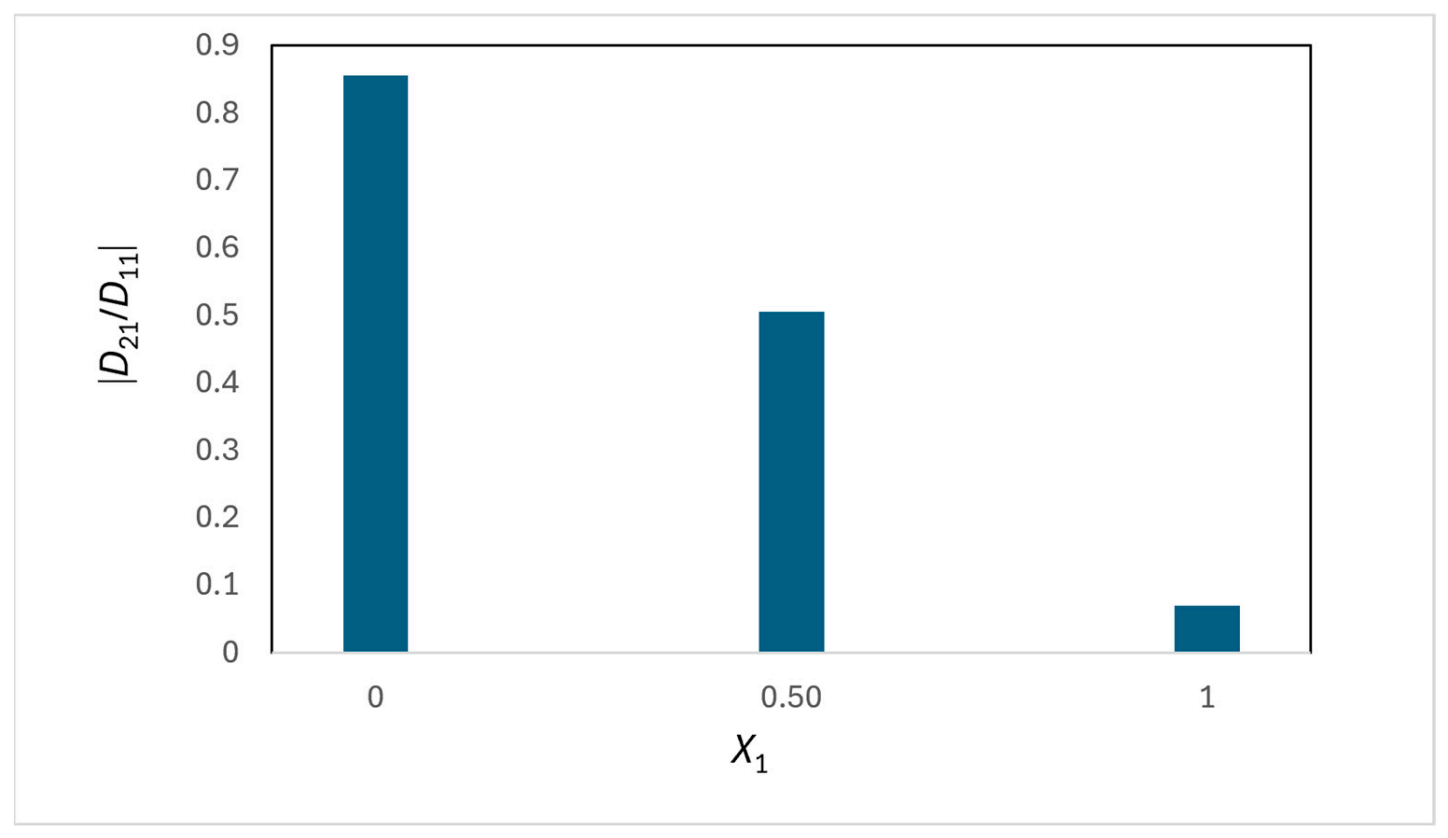
Estimation of moles of Na_4_PRA (component 2) transported for each mol of E·(HCl)_2_ (component 1) for different values of molar fraction, *X*_1_:|*D*_21_/*D*_11_| represents the number of moles of Na_4_PRA counter-transported for each mol of E·(HCl)_2_.

Coupled diffusion in these systems can be analyzed using the calculated $D_{12}/D_{22}$ (Figure 3) and $D_{21}/D_{11}$ (Figure 4) ratios for the aqueous INH + ${\mathrm{Na}}_{4}\mathrm{PRA}$ system, as well as *D*_21_/*D*_11_ (Figure 5) for (E·(HCl)_2_/Na_4_PRA.

These ratios enable us to determine the number of moles of one of the components transported per mole of the other.

Based on *D*_12_/*D*_22_ ratios (Figure 3), one mole of diffusing Na_4_PRA co-transports up to 2.5 moles of INH. Furthermore, the *D*_21_/*D*_11_ ratios (Figure 4) indicate that one mole of diffusing INH counter-transports a maximum of 0.4 moles of Na_4_PRA.

As indicated by the *D*_21_/*D*_11_ data in Figure 5, the diffusion of one mole of E·(HCl)_2_ drives the counter-transport of up to roughly 0.9 moles of Na_4_PRA.

The observed coupled diffusion may be explained by potential 1:1 supramolecular complexation. Support for this came from ^1^H NMR spectroscopy, which demonstrated the formation of 1:1 ($\mathrm{INH}:{\mathrm{Na}}_{4}\mathrm{Pra}$) and ($E\cdot {\left(\mathrm{HCl}\right)}_{2}:{\mathrm{Na}}_{4}\mathrm{Pra}$) complexes, with $\log\left(K_{f}\right)$ values of $0.62\left(\pm 0.08\right)$ for isoniazid (INH) and $4.72\left(\pm 0.10\right)$ for ethambutol, respectively.

## 4. Materials and Methods

### 4.1. Materials

Table 4 indicates all reagents, which were used as received, in the present work. All these compounds were used without further purification. The solutions for the diffusion measurements were prepared using Millipore-Q water (specific resistance = 1.82 × 10^5^ Ω m, at 298.15 K). Solutions for NMR measurements were prepared in D_2_O. All solutions were freshly prepared at 298.15 K before each experiment.

### 4.2. Synthesis of Resorcinarenes

#### 4.2.1. Synthesis of *C*-Tetra(propyl)calix[4]resorcinarene (**1**)

A resorcinol solution (10 mmol) in 20 mL of ethanol:water (50%) mixture was added dropwise to 1.0 mL of concentrated HCl, and then 10 mmol of butanal was added dropwise. The mixture was refluxed for 6 h, and the compound was precipitated and filtered with water, producing a solid product, which was purified via recrystallization in ethanol and characterized by means of IR, ^1^H-NMR, and ^13^C-NMR.

*C*-tetra(propyl)calix[4]resorcinarene (**1**): Yield of 75%; IR (cm^−1^): 1280 (C-O), 2980 (C-H), 3068 (ArC-H), 3280 (ArO-H); ^1^H-NMR, DMSO-d_6_, δ (ppm): 0.90 (t, 12H, CH_3_), 1.19 (m, 8H, CH_2_), 2.07 (m, 8H, CH_2_), 4,23 (t, 4H, CH), 6.15 (s, 4H, ArH), 7,24 (s, 4H, ArH), 8.94 (s, 8H, OH). ^13^C-NMR, DMSO-d_6_ δ (ppm): 14.4; 21.1; 33.0; 36.1; 102.8; 123.2; 125.5; 152.0.

#### 4.2.2. Synthesis of Tetrasodium-5,11,17,23-tetrakissulfonatemethylen-2,8,14,20-*C*-tetra(propyl)calix[4]resorcinarene (**2**)

0.2 mol of *C*-tetra(propyl)calix[4]resorcinareno (**1**) was reacted with a solution of 1.0 mol of formaldehyde (37%) and 1.0 mol of sodium sulfite in water (30 mL), which was refluxed at 90–95 °C for 4 h. Dilute hydrochloric acid was added after cooling up to pH 7, and then acetone was added in order to precipitate products **2**. The solids were filtered, washed with acetone, and vacuum-dried.

*Tetrasodium-5,11,17,23-tetrakissulfonatemethylen-2,8,14,20-C-tetra(propyl)calix[4]resorcinarene (***2***)*: Yield of 26.5%, IR (cm^−1^): 1051 (S=O), 2950 (CH), 3012 (ArCH), 3414 (broad, O-H). ^1^H-NMR, D_2_O, δ (ppm): 0.96 (t, 12H, CH_3_), 1.59 (m, 8H CH_2_), 2.23 (m, 8H, CH_2_), 4.20 (s, 8H, Ar-CH_2_-SO_3_Na), 4.45 (t, 4H, CH), 7.23 (s, 4H, ArH); ^13^C-NMR, D_2_O, δ (ppm): 14.1, 22.3, 27.9, 34.3, 48.3, 109.5, 123.1, 124.9, 150.1. LC ESI–TOF/MS analysis showed a signal at *m*/*z* = 1121.4193 corresponding to [M+H]^+^, Calcd. for C_44_H_52_Na_4_O_20_S_4_, *m*/*z* = 1121.15 corresponding to [M+H]^+^.

### 4.3. pH Measurements

The pH of the solutions was measured using a Radiometer PHM 240 pH meter (Radiometer Analytical SAS, Villeurbanne, France) equipped with an Ingold U457-K7 combined pH electrode; pH was measured in fresh solutions, and the electrode was calibrated immediately before measuring each set of solutions using the IUPAC-recommended pH 4.0, 7.0, and 10.0 buffers. From the pH meter calibration, a zero pH of 6.09 ± 0.05 and sensitivity higher than 98.5% were obtained.

### 4.4. NMR Measurements

For the studies, a total of six samples were prepared, of which three were reference samples: isoniazid (INH), ethambutol (E·(HCl)_2_), and sulfomethylated resorcinarene (**2**), each sample dissolved in 700 μL of deuterated water. The remaining three samples consisted of an equimolar mixture of INH with E·(HCl)_2_), INH with resorcinarene 2 and E·(HCl)_2_ with 2, in the same way each sample dissolved in 700 μL of deuterated water.

^1^H-NMR titrations were recorded in D_2_O at 400 MHz using a Bruker Avance 400 instrument (Bruker Scientific Instruments, Billerica, MA, USA). Chemical shifts are reported in ppm (measurement error of chemical shift 0.010), using the residual solvent signal (D_2_O) as a reference. To determine the stoichiometry of the complex, the molar ratio method was used for both isoniazid (INH) and ethambutol (E·(HCl)_2_) with sulfomethylated resorcinarene (**2**). For example, in the latter case, a total of seven samples of mixtures of 2 and E·(HCl)_2_ were prepared using a fixed amount of resorcinarene **2** (30 mg in 700 μL), and a solution of E·(HCl)_2_ was added until a 1:1 molar ratio was reached. The total volume of solution prepared was, in all cases, 700 μL. After each addition, the ^1^H-NMR spectrum was recorded. The concentrations of the guest and host in the solution were corrected for each addition. Chemical shift values of the proton signal at 3.56 ppm of E·(HCl)_2_, which is the most affected in the mixtures, are shown in Table 5.

For both systems, a 1:1 host–guest stoichiometry was established. An equilibrium model was subsequently proposed, and the binding constants (${\log K}_{f}$) for each complex were determined using the HypNMR2008 program (payment software). For this purpose, the chemical shift values of the proton signal most significantly affected in the mixtures with resorcinarene **2** were used. The resulting ${\log K}_{f}$ values were 4.72 for ethambutol and 0.62 for isoniazid (INH), respectively. The fit was performed using the nonlinear least-squares method, with σ = 1.71 and σ = 0.009153, respectively.

### 4.5. Diffusion Measurements

#### 4.5.1. Ternary Diffusion Coefficients in Aqueous INH(1)+Na_4_PRA(2) Solutions

Ternary mutual diffusion coefficients for aqueous INH (component 1) + Na_4_PRA (component 2) solutions were measured at different concentrations using the Taylor dispersion technique [34,35,36,37,38,39].

Succinctly, this method consists of dispersing small amounts of solution injected into laminar carrier streams of solution of different compositions flowing through a long capillary tube. At the start of each run, a 6-port Teflon injection valve (Rheodyne, model 5020, Rheodyne, LLC, Rohnert Park, CA, USA) was used to introduce 0.063 cm^3^ of solution into a laminar carrier stream of slightly different composition. A flow rate of 0.23 cm^3^ min^−1^ was maintained by a metering pump (Gilson model Minipuls 3, Gilson, Inc., Middleton, WI, USA) to give retention times of about 8000 s. The dispersion tube (length 3279.9 (±0.1) cm, and internal radius 0.0322 ± 0.00003 cm) and the injection valve were kept at 298.15 (±0.01) K in an air thermostat. Dispersion of the injected samples was monitored using a differential refractometer (Waters model 2410, Waters Corporation, Milford, MA, USA) at the outlet of the dispersion tube. Detector voltages, *V*(*t*), were measured at 5 s intervals with a digital voltmeter (Agilent 34401 A, Agilent Technologies, Santa Clara, CA, USA).

Evaluation of the binary diffusion coefficients (D) defined by Fick’s relation (Equation (1)) was performed by fitting the dispersion profile (Equation (2)) to the recorded detector voltage data. The parameters optimized in this fit include the baseline voltage ($V_{0}$), baseline slope ($V_{1}$), peak height ($V_{\max}$), and the mean sample retention time ($t_{r}$).

*J* = −*D*∇*C* (1)

(2) $$V(t)=V_{0}+V_{1}t+V_{max}{\left(\frac{t_{r}}{t}\right)}^{1/2}exp\left[-\frac{12D(t-t_{r}{)}^{2}}{r^{2}t}\right]$$

*J* is the molar flux of component 1 driven by the concentration gradients ∇*c*.

The ternary diffusion behavior of aqueous ternary systems containing the drug INH (component 1) and Na_4_PRA (component 2) was evaluated using the ternary diffusion equations (Equations (3) and (4)):

*J*_1_ = −*D*_11_∇*c*_1_ − *D*_12_∇*c*_2_ (3)

*J*_2_ = −*D*_21_∇*c*_1_ − *D*_22_∇*c*_2_ (4)

*J*_1_ and *J*_2_ represent the molar fluxes of INH (1) and Na_4_PRA (2) driven by the concentration gradients ∇*c*_1_ and ∇*c*_2_ of each solute 1 and 2, respectively. Main diffusion coefficients *D*_11_ and *D*_22_ give the flux of each solute driven by its own concentration gradient. Cross-diffusion coefficients *D*_12_ and *D*_21_ give the coupled flux of each solute driven by a concentration gradient in the other solute. A negative *D_ik_* coefficient indicates counter-current coupled transport of solute *i* from regions of lower to higher concentration of solute *k*. A positive *D_ik_* cross-coefficient (*i* ≠ *k*) indicates co-current coupled transport of solute *i* from regions of higher to lower concentrations of solute *k*.

The ternary mutual diffusion coefficients (*D*_ik_) were obtained by fitting the ternary dispersion equation (Equation (5)) to at least two replicate peak pairs for each carrier stream.

(5) $$V\left(t\right)=V_{0}+V_{1}t+V_{max}{\left(t_{r}/t\right)}^{1/2}\left[W_{1}exp\left(-\frac{12D_{1}{\left(t-t_{r}\right)}^{2}}{r^{2}t}\right)+(1\;-W_{1})exp\left(-\frac{12D_{2}{\left(t-t_{r}\right)}^{2}}{r^{2}}\right)\right]$$

In Equation (5), $D_{1}\;and\;D_{2}$ represent the eigenvalues of the ternary $D_{ik}$ matrix; $V_{0}$, $V_{1}$, and $V_{\max}$ denote the baseline voltage, baseline slope, and peak height; $t_{r}$ and r are the retention time and tube radius; and $W_{1}$ and $1-W_{1}$ are the normalized pre-exponential factors.

In these experiments, small volumes of ∆V of solution containing INH (1) +Na_4_PRA (2) at concentrations of *C*_1_ + Δ*C*_1_, *C*_2_ + ΔC_2_ are injected into carrier solutions of composition *C*_1_ and *C*_2_.

#### 4.5.2. Ternary Diffusion Coefficients in Aqueous E·(HCl)_2_ (1) + Na_4_PRA (2) Solutions

The diffusion behavior of aqueous ternary systems containing E·(HCl)_2_ (component 1) and Na_4_PRA (component 2) was also evaluated by using Taylor dispersion. As a first approximation, diffusion in these systems may be governed by the coupled ternary Fick Equations (5) and (6). However, it must be emphasized that treating the parameters measured in our laboratory within the volume-fixed reference frame as ternary diffusion coefficients is an approach, given that these solutions do not constitute true ternary systems possessing more than two independent components. In aqueous E·(HCl) _2_ (1) + Na_4_PRA (2) solutions, there are fluxes of four different ions (EH_2_^2+^, Cl^−^, Na^+^, PRA^4−^) constrained only by electroneutrality. As a result, there are three independent mutual diffusion fluxes. For example, because Na^+^ and PRA^4−^ ions have different mobilities, they are not required to diffuse at the same speed along Na_4_PRA concentration gradients in E·(HCl)_2_ (1) + Na_4_PRA (2) solutions. The smaller, more mobile Na^+^ will diffuse at a higher speed than the PRA^4−^ ions. The excess flux of Na^+^ ions relative to PRA^4−^ ions cannot be accounted for by the flux *J*_1_ of E·(HCl)_2_ (1). Similarly, the Na^+^ and PRA^4−^ ions diffuse at different speeds along E·(HCl)_2_ concentration gradients, which is not described by the flux *J*_2_ of Na_4_PRA (2). For E·(HCl)_2_ (1) + Na_4_PRA (2) solutions, this means

*J*_1_ E·(HCl)_2_ ≠ −*D*_11_∇*C*_1_ − *D*_12_∇*C*_2_ (6)

*J*_2_ (Na_4_PRA) ≠ −*D*_21_∇*C*_1_ − *D*_22_∇*C*_2_ (7)

To describe diffusion in E·(HCl)_2_ (1) +Na_4_PRA (2) solutions, the flux of a third electrolyte must also be specified. We will choose NaCl as the third electrolyte, which gives the quaternary Fick equations:

*J*_1_E·(HCl)_2_ = *D*_11_∇*C*_1_ − *D*_12_∇*C*_2_ − *D*_13_∇*C*_3_ (8)

*J*_2_(Na_4_PRA) = *D*_21_∇*C*_1_ − *D*_22_∇*C*_2_ − *D*_23_∇*C*_3_ (9)

*J*_3_(NaCl) = *D*_31_∇*C*_1_ − *D*_32_∇*C*_2_ − *D*_33_∇*C*_3_ (10)

In practice, measuring the nine quaternary *D*_ik_ coefficients is exceedingly difficult, particularly for the present system. Moreover, no existing theory can reliably predict these coefficients with the precision demanded by science and industry. To deal with these problems, we have measured ternary diffusion coefficients (*D*_ik_) for E·(HCl)_2_ (*C*_1_) + Na_4_PRA(*C*_2_) solutions by assuming that the flux of NaCl (*C*_3_), the third electrolyte, is negligible.

The ternary dispersion profiles for these solutions E·(HCl)_2,_ component 1 + Na_4_PRA, component 2 were prepared by injecting solution samples of E·(HCl)_2_ (1) or Na_4_PRA (2), with compositions *C*_1_ + ∆*C*_1_ and *C*_2_ + ∆*C*_2_, into flow solutions of composition *C*_1_+ *C*_2_. The ternary diffusion coefficients (*D*_11_, *D*_22_, *D*_12_ and *D*_21_) were evaluated by fitting the ternary dispersion described by Equations (8) and (9) and were determined by fitting Equation (7) to the experimental dispersion profiles [39,40].

*D*_1_ and *D*_2_ are the eigenvalues of the 2 × 2 *D*_ik_ matrix of ternary diffusion coefficients. (Equations (13) and (14)) and *α*_1_ is the fraction of the initial refractive index difference due to E·(HCl)_2_. *R*_1_ and *R*_2_ are the detector sensitivities for E·(HCl)_2_ (1) and Na_4_PRA (2): *R*_1_ = ∂*V*/∂*C*_1_ and *R*_2_ = ∂*V*/∂*C*_2_.

(11) $$D_{1}=\left\{D_{11}+D_{22}+\left(D_{11}-D_{22}\right)\sqrt{1+\left[4D_{12}D_{21}/{\left(D_{11}-D_{22}\right)}^{2}\right]}\right\}/2$$

(12) $$D_{2}=\left\{D_{11}+D_{22}-\left(D_{11}-D_{22}\right)\sqrt{1+\left[4D_{12}D_{21}/{\left(D_{11}-D_{22}\right)}^{2}\right]}\right\}/2$$

Ternary mutual diffusion coefficients were calculated from the *D*_1_, *D*_2_, *a*, *b* fitting parameters and the relative detector sensitivity *R*_2_/*R*_1_ using

(13) $$D_{11}=D_{1}+\frac{\;a\left(1-a-b\right)}{b}\left(D_{1}-{\;D}_{2}\right)$$

(14) $$D_{12}=\frac{R_{2}}{R_{1}}\;\frac{\;a\;\left(1-a\right)}{b}\left(D_{1}-{\;D}_{2}\right)$$

(15) $$D_{21}=\frac{R_{1}}{R_{2}}\;\frac{\;\left(a+b\right)\left(1-a-b\right)}{b}\left(D_{2}-D_{1}\right)$$

(16) $$D_{22}={\;D}_{2}+\frac{\;a\left(1-a-b\right)}{b}\left(D_{2}-D_{1}\right)$$

The *a* and *b* parameters in these Equations (15)–(18) are given by

(17) $$a=\frac{D_{11}{-D}_{1}-\left(R_{1}/R_{2}\right)D_{12}}{D_{2}-D_{1}}$$

(18) $$b\;\;=\;\;\frac{D_{22}-D_{11}+(R_{1}/R_{2})D_{12}-(R_{2}/R_{1})D_{21}}{D_{2}-D_{1}}$$

## 5. Conclusions

The multicomponent diffusion coefficients ($D_{11}$, $D_{12}$, $D_{21}$, and $D_{22}$) of aqueous INH/${\mathrm{Na}}_{4}\mathrm{PRA}$ and E·(HCl)_2_/${\mathrm{Na}}_{4}\mathrm{PRA}$ systems were determined at 298.15 K. Significant coupled diffusion was observed between these drugs and ${\mathrm{Na}}_{4}\mathrm{PRA}$, particularly in the case of E·(HCl)_2_. This confirms that the macrocyclic host strongly influences solute transport in solution due to non-negligible intercomponent interactions. Furthermore, the diffusion data indicate the formation of supramolecular host–guest complexes. This is corroborated by NMR evidence showing the inclusion of both INH and $E\cdot {\left(\mathrm{HCl}\right)}_{2}$ within the resorcinarene cavity. The findings presented here provide the fundamental transport data required to model diffusion profiles for future pharmaceutical applications.

These insights strongly suggest that ${\mathrm{Na}}_{4}\mathrm{PRA}$ holds great potential as an effective drug carrier.

## Figures and Tables

**Table 1:** Mean diffusion coefficients (*D*_lit_) and the respective standard deviations of the means ^a^, *S*_D_, for 0.100 ${\mathrm{mol}\;\mathrm{dm}}^{-3}$ KCl.

*D* ± *S*_D_ ^c^	*D*_lit_/(10^−9^ m^2^ s^−1^)
1.840 ± 0.007	1.846 ^b^
	1.849 ^c^

^a^ Averaged result for *n* = 6 experiments; ^b,c^ Values obtained by interpolation from data reported in references [32,33].

**Table 2:** Ternary mutual diffusion coefficients (*D*_11_, *D*_12_, *D*_22_, *D*_21_) of INH (*C*_1_) + Na_4_PRA (*C*_2_) and the respective standard deviations (*S_D_*) in aqueous solutions at 298.15 K.

*C*_1_ ^a^	*C*_2_ ^a^	*X*_1_ ^b^	*D*_11_ ± *S*_D_ ^c^	*D*_12_ ± *S*_D_ ^c^	*D*_21_ ± *S*_D_ ^c^	*D*_22_ ± *S*_D_ ^c^
0.0000	0.0100	0.00	1.080 ± 0.015	0.035 ± 0.028	−0.409 ± 0.002	0.600 ± 0.020
0.0025	0.0075	0.25	1.070 ± 0.010	0.390 ± 0.028	−0.030 ± 0.002	0.580 ± 0.010
0.0050	0.0050	0.50	1.063 ± 0.016	0.825 ± 0.050	−0.045 ± 0.020	0.586 ± 0.020
0.0075	0.0025	0.75	0.989 ± 0.016	1.020 ± 0.050	−0.015 ± 0.020	0.614 ± 0.020
0.0100	0.0000	1.00	0.850 ± 0.022	1.592 ± 0.090	0.005 ± 0.002	0.626 ± 0.020

^a^ *C*_1_/(mol dm^−3^) and *C*_2_/(mol dm^−3^); ^b^ *X*_1_ represents the mole fraction of INH (*X*_1_ = C_1_/(*C*_1_ *+* C_2_); ^c^ *D*_ik_ ± *S*_D_/(10^−9^ m^2^ s^−1^) represent the mean values obtained from six independent experiments. Considering that u is the standard uncertainty and *u*r is the relative standard uncertainty, standard uncertainties are u(*C*) = 1.0·10^−3^ mol dm^−3^ (max); u(*T*) = 0.01 K; u(*P*) = 2.03 kPa; *u*(*T*) = 0.01 K; *u*(*D*) = 0.01 × 10^−9^ m^2^ s^−1^.

**Table 3:** Ternary mutual diffusion coefficients ^a^ (*D*_11_, *D*_12_, *D*_22_, *D*_21_) of E·(HCl)_2_ (*C*_1_) + Na_4_PRA (*C*_2_) and the respective standard deviations, *S_D_*, in aqueous solutions at 298.15 K.

*C*_1_ ^a^	*C*_2_ ^a^	*X*_1_ ^b^	*D*_11_ ± *S* ^c^	*D*_12_ ± *S*_D_ ^c^	*D*_21_ ± *S*_D_ ^c^	*D*_22_ ± *S*_D_ ^c^
0.000	0.010	0.0	0.817 ± 0.009	0.003 ± 0.008	−0.700 ± 0.004	0.599 ± 0.007
0.005	0.005	0.5	0.890 ± 0.015	0.009 ± 0.020	−0.450 ± 0.020	0.560 ± 0.010
0.010	0.000	1.0	0.950 ± 0.010	0.053 ± 0.020	−0.065 ± 0.040	0.541 ± 0.011

^a^ *C*_1_/(mol dm^−3^) and *C*_2_/(mol dm^−3^); ^b^ *X*_1_ represents the mole fraction of E·(HCl)_2_ (*X*_1_ = *C*_1_/(*C*_1_ *+ C*_2_); ^c^ *D*_ik_ ± *S*_D_/(10^−9^ m^2^ s^−1^) represent the mean values obtained from six independent experiments. Considering that *u* is the standard uncertainty and *u*r is the relative standard uncertainty, standard uncertainties are u(*C*) = 1.0·10^−3^ mol dm^−3^ (max); u(*T*) = 0.01 K; u(*P*) = 2.03 kPa; *u*(*T*) = 0.01 K; *u(D*) = 0.01 × 10^−9^ m^2^ s^−1^.

**Table 4:** Sample description.

Chemical Name	Source ^a^	CAS Number	Mass Fraction Purity
Isoniazid (INH)	Sigma-Aldrich	54–85–3	>0.99
Ethambutol (E·(HCl)_2_ *C*-tetra(propyl)resorcin[4]tetrasulfonate (Na_4_PRA)	Sigma-Aldrich-	1070–11–7-	>0.99
D_2_O	Sigma-Aldrich	7789-20-0	>0.99
H_2_O	Millipore-Q water (*κ* = 1.82 × 10^5^ Ω m at 298.15 K)	7732–18-5	

^a^ The mass fraction purity is on a water-free basis; these data are provided by the suppliers.

**Table 5:** Chemical shift values of proton signals, obtained by ^1^H-NMR, as a function of the molar ratio between Resorcinarene **2** and E·(HCl)_2_.

Guest/(2)	E·(HCl)_2_ δ (ppm)	INHδ (ppm)
0.0	3.56	7.68
0.2	2.88	7.64
0.4	2.84	7.60
0.6	2.79	7.57
0.8	2.77	7.54
1.0	2.64	7.53
2.0	2.63	7.52

## Data Availability

The original contributions presented in this study are included in the article. Further inquiries can be directed to the corresponding authors.
